# Surgical Cases Reported to the Louisville Medico-Chirurgical Society*Stenographically reported for this journal by C. C. Mapes, Louisville, Ky.

**Published:** 1898-06

**Authors:** William L. Rodman

**Affiliations:** Professor of Surgery and Clinical Surgery in the Kentucky School of Medicine; Surgeon to the Kentucky School of Medicine Hospital; Surgeon to the Sts. Mary and Elizabeth Hospital; Consulting Surgeon to the Louisville City Hospital, etc., Louisville, Ky.


### Southern Medical Record.

A MONTHLY JOURNAL OF MEDICINE AND SURGERY.

VOL. XXVIII. ATLANTA, GA., JUNE, 1898. NO. 6.

### Original Articles.

### SURGICAL CASES REPORTED TO THE LOUISVILLE MEDICO-CHIRURGICAL SOCIETY.*

By WILLIAM L. RODMAN, A.M., M.D.

Professor of Surgery and Clinical Surgery in the Kentucky School of Medicine; Surgeon to the Kentucky School of Medicine Hospital; Surgeon to the Sts. Mary and Elizabeth Hospital; Consulting Surgeon to the Louisville City Hospital, etc., Louisville, Ky.

The first patient, D. W., male, aged thirty-five years, I am able to show you through the courtesy of Professor James M. Holloway. It is a very interesting case of multiple enchondroma, and I think several of the gentlemen present assisted in one or more of the operations that have been performed upon him. I also present for your inspection several photographs showing the patient's condition at different times, and a skiagraph of his right arm before an atypical resection of the elbow last summer. Unfortunately, Doctor Holloway is unable to be present this evening; he was to have brought a photograph giving an accurate idea of this man's condition before the first operation, but I have found an exact copy of it in Park's "Surgery."

As above stated, the case is one of multiple enchondroma, and is really one of the most remarkable on record. I do not know of a case more interesting than this, unless it be the celebrated case of Steudel, which is represented in nearly all of our works on surgery. The cut in Park's "Surgery" shows the man before any of the operations were performed upon him; the different photographs show his condition after the first, second, third, fourth and fifth operations.

Dr. A. M. Cartledge: I remember this case distinctly, and this is the first time I have seen the patient since the first operation, which was a synchronous triple amputation. It was a

*Stenographically reported for this journal by C. C. Mapes, Louisville, Ky.

very extensive case of multiple enchondroma involving the hands and feet originally. The first operation was performed several years ago. The patient was under the care of Doctor James M. Holloway, who invited a number of his surgical friends to assist him in the operation. I believe I amputated a portion of the left foot, and some of the other gentlemen present operated upon the hands, removing several of the fingers which contained masses of enchondromatous tissue as large as potatoes. The patient at that time presented some enchondromatous masses about the shoulder and upper part of the humerus which it was not deemed advisable to attack surgically. I am a little astonished that the patient is able to be here to-night; that he has lived so long after such extensive operative interference.

DR. W. L. RODMAN: While these tumors were originally benign—undoubtedly enchondromatous in their nature—the last examination made, I think, during the past summer when an atypical resection of the elbow joint was performed, was submitted to the microscopist and pronounced to be sarcoma, showing that degeneration of the enchondroma into sarcoma had already taken place. As the patient has now been excused, I will say that Doctor Holloway is going to amputate the right arm to-morrow morning, because of the enormous sarcomatous enlargement involving the elbow. I think the original operations upon this man were performed five years ago.

*Case 2.* This patient, Mr. H., you will remember as having been before this society on a previous occasion, just prior to an operation upon the right leg. He had at that time a very much enlarged condition of the knee, an enlarged inner condyle, evidently tuberculous in character. Prior to that time I had gone into a number of the other joints; I had removed the entire sterno-clavicular joint upon both sides; I had gone into both shoulder joints and resected the upper part of the humerus; I had also removed the spine of the scapula on the left side, also a part of one of the ribs; and the last operation was done upon the knee a year ago last month. His general condition at the time was far from good, yet the result of the rather extensive surgery has been most excellent. Notwithstanding the fact that I went into the joint and removed the entire inner condyle, he has recovered without a stiff knee; you will observe that he walks freely and without limp. His general condition has greatly improved within the last year. He has a tubercular history in the family, but I think at the present time his lungs are entirely clear.

*Case 3.* The third case is also a very interesting one; it is a compound fracture of the middle of the thigh, which occurred in Indiana last July. The wound became infected and the man was afterwards brought to this city to me, and we had a desperate fight to preserve the leg. We thought for a long time that he would lose his leg on account of the very extensive fracture and the fact that it was suppurating so freely when he came here and continued to do so for a long time afterwards. We had to go into it a number of times and remove pieces of dead bone; then afterwards the parts were immobilized in plaster, and I think we have secured a very good union for a compound fracture of the femur. Motion at the present time seems to be very much restricted at the knee on account of the long encasement in plaster, but since the plaster was removed last Monday there has been some improvement and I doubt not motion will speedily return.

I have had few more interesting cases than this. When the man was first brought here it was thought the leg would have to be amputated, and we also felt that the patient might lose his life on account of the extensive suppuration. I feel very much gratified at the outcome. Had he been less robust I think he would not only have lost his leg but lost his life as well.

Union was very much delayed; it was four months after the fracture before there was any attempt at union. There is not more than three-fourths of an inch of shortening. I anticipated that there would be at least two and a half or three inches shortening, on account of the oblique nature of the fracture, and am really surprised that shortening is not much greater than it is.

I removed the sequestra of bone, freshened the edges with pliers, rubbing the ends of the bone together freely, then held them in apposition by means of a plaster dressing.

The patient was brought to me one month after the injury.

DR. ERNEST LAPLACE, of Philadelphia (present by invitation): The case of multiple enchondromata shown by Doctor Rodman is certainly one of the most remarkable I have ever seen, especially from its etiological standpoint. The great problem underlying the development of sarcoma is far from being solved. There is evidently something which enters the body, develops there, and while developing forces a commensurate development of new tissue. We do not know its nature or the peculiarities of its development. This irritant does not develop a toxin virulent enough to destroy the cells which it brings into existence. Very different is it from the action of the streptococcus pyogenes areus.

By way of explanation, I would say that apparently a strange paradox exists in pathology, viz.: some virulent germs produce innocent diseases, and vice versa. The most innocent disease we know is perhaps an abscess. As soon as the streptococcus develops, it produces a toxin which kills immediately the white blood corpuscles, and they undergo fatty degeneration, resulting in pus or the formation of an abscess.

Would that the living organism that may be the cause of sarcoma were as virulent as the streptococcus pyogenes and some of the other germs, for then instead of having a mass of new tissue we would have the formation of pus. Unfortunately that germ is not virulent enough; it develop[illegible]stantly, and under this constant irritation a constant p[illegible]feration process goes on within the tissue and accumulates there as a mass, and that mass forms such a tumor as has just been before us, the so-called round-cell sarcoma.

Through the courtesy of my friend, Doctor Rodman, I had an opportunity this morning to examine this man, and putting my hand on the swelling at the elbow the thought occurred to me that it might be an abscess, so soft and fluctuating seemed the mass. Doctor Rodman afterwards told me the previous history of the case; that it had been a case of multiple enchondroma, etc. I am more than thankful for this opportunity to bring out this thought, that a man's system is such that it is capable of undergoing a peculiar change under the influence of a pathological process. Now this man at some time during his life developed a peculiar diathesis by which the cartilages, above all other portions of the body, became the seat of new tissue development. It matters not whether the growth be an osteoma or an enchondroma as far as the original disease is concerned; it is practically the same thing; it is always the fibrous tissue that is at fault. Therefore I imagine at first this man developed a disease not unlike that which we find in children called osteo-myelitis of adolescence, where through the whole body small abscesses form within the bones because of infection by the streptococcus pyogenes. In this man a similar condition occurred, except that the infection was not as virulent as from the streptococcus pyogenes, because that organism would have destroyed the cells and we would have had the formation of pus instead of an enchondromatous mass. It developed slowly, and as the cells grew produced a mass, or it may be many masses, of imperfect cartilage cells. We have an analogous condition in the so-called multiple fibromata, where, instead of the cartilage being developed, we have a number of fibromate developed all over the body. I remember a man who had at least fifty of these fibromata scattered over the body.

When an operation is performed and these tumors are removed, we do not in any way improve the character of the soil. Shock lessens the vitality of the patient, and whereas he was previously a suited subject for the development of this particular disease, he is now more suited; in other words, it is the common experience of all of us in removing malignant tumors, that after the growth is removed it often returns with doubled activity. In this man's original condition the tumors were fibrous and hard; now the tumor present is very soft. It grows faster and faster simply because of lessened vitality of the soil.

Nothing could be a better lesson to us than this case to bring out the clinical fact, that all tumors, no matter how benign they may seem, should be removed. These tumors have a great tendency to undergo the so-called sarcomatous degeneration, w[illegible] term is not suited to the modern notions of the case, in[illegible]uch as we do not deal with a growth which undergoes degeneration, but the sarcomatous condition is simply an expression of the same old growth returning, developing so rapidly that instead of the cells having had time to go on to maturity or fully formed fibrous cells, they grow so rapidly that they are found in their adolescence and a soft mass is the result. This feature is aptly illustrated by the case which has been before us; whereas this man had hard growths in the beginning of his trouble, the one which is present to-day seems to be as soft as an abscess. The cells are of the granulation or round variety; the oldest ones have reached a degree of spindle shape, and as a result there is absence of hardness which we would expect in an ordinary fibroma. Therefore I feel very confident that when this tumor is removed and submitted for examination a large mass of round cells will be found which is one of the points of differentiation between rapidly developing growths and those which grow slowly, and if we knew nothing of the circumstances would say at once this specimen came from a growth which developed very rapidly because it is made up principally of round cells.

Another way of illustrating the subject which I have used several times is simply this: You can judge whether a village is growing rapidly in its population, or whether it is at a standstill, by observing the number of children. If you go on the street and see only grown people, you say the town is not developing; if, on the contrary, you see a great many children, you say it is a rapidly developing place. When the pathologist submits a specimen of a tumor like this to microscopical examination and sees all round cells, he will tell you without knowing anything further about the case, that the tumor is a rapidly developing round-cell sarcoma. If, on the contrary, he had one of the first tumors removed from this patient made up of hard fibrous tissue, he would tell you that it was a tumor which had developed slowly.

The only point I desire to bring forward is this: I believe that the rapidity of a growth, its malignancy, and the slowness of its growth, its benignity, depend not so much upon the seed as upon the soil; and I maintain that whereas the seed is the same the growth depends upon the nature of the soil; that a soil which is little suited to development at first as time goes on becomes more suited, the growth develops more rapidly, showing the varios characteristics of the sarcomata—fibrous cells, spindle-shaped cells, and round cells.

DR. CARL WEIDNER: I can hardly add anything to the excellent exposition which Doctor Laplace has given us, and we all probably agree with him in the explanation offered as to the histological development of sarcomatous tumors, and the occasional change in character of growths from benign to malignant. We do not know anything about the etiological factor of sarcoma; we are totally ignorant as to whether it is a micro-organism that spends its force here—and the chances are more in favor of that than anything else—or whether there is some condition of the system now unknown to us. I think surgeons have recognized more and more that it is the safest plan to remove thoroughly and early any growth with which they meet. The previous speaker has given us such a beautiful explanation of the histological development of the growths under consideration that I can add nothing.

The idea advanced by him concerning the relative effects of the more virulent organisms and those of the less virulent ones is a very pretty one, and is in accordance with clinical facts. Unfortunately we do not know anything about the morbid agent in sarcoma. Whatever this unknown agent may be, however, it must be something that spends its force slowly and is fought by the organism for sufficient length of time to enable the individual cell to resist its irritation so that it may still live, while with the more virulent toxins of the pyogenic organisms the cell is rapidly destroyed and converted into masses entirely foreign to the body.

I am not personally aware of the changes which have taken place in the case reported. Since seeing the patient here and the photographs I remember having been present at the last operation done by Professor Holloway on the elbow, and remember that the Doctor laid stress upon the softened cystic condition of the tissues, which he ascribed to the formation of a gelatinous substance within them. We know that any tumor is liable to undergo various degenerations, such as colloid, mucoid, etc. I do not know whether the previously removed tumors showed any signs of malignancy, as I do not remember to have made the examination.

DR. A. M. CARTLEDGE: We are all indebted to Doctor Laplace for the excellent exposition of the subject which he has given us. Certainly in the entire range of pathology we meet with nothing of so much interest at the present day as the subject of sarcoma. This disease has always been to me one of the greatest interest. It occupies a mid-position, and the facts brought out by Doctor Laplace suggest others, and certainly we have all been interested in them His remarks suggest the correlation of germs in a marked way. To go a little further back and elaborate one point, assuming, as he says, something we do not know positively, and to reason from analogy, the similarity between sarcoma and the granulatoma is one of the most striking in pathology; and while we have had very little upon which to base the opinion that carcinoma is an infectious disease, we have many reasons to believe that the sarcomas are infectious or germ diseases. Certainly similar tissue changes in a measure take place in sarcoma as take place in tuberculous processes, in actinimicosis and in syphilis. I was especially struck with what Doctor Laplace said in regard to the organism of sarcoma; it is a beautiful theory, and one that has a practical bearing upon the etiology of the malignant and benign growths. Like the Doctor, I do not believe in true sarcomatous degeneration; it is a mistake to make a statement of this kind. We often speak of an enchondroma or any innocent growth undergoing sarcomatous degeneration, which I believe is based upon a wrong conception. My idea is that there is not a degeneration but a secondary infection; the soil is prepared by a more innocent influence, and then there occurs a secondary infection; and certainly we have many reasons to believe that sarcoma is an infectious or germ disease. We must realize that benign growths create a favorable soil for the development of malignant tumors.

The Doctor spoke of the primary slow development of a sarcoma; then as the soil becomes better suited there is a rapid infiltration and dissemination of the disease. I have been impressed with this fact in two recent cases which I have prominently in my mind. Sarcoma may sometimes last a long time- of a fairly virulent type; then we may have a rapid develop, ment coming on rather suddenly, with the formation of large nodules and other well-known characteristics, showing that the soil becomes more favorable for the development of the disease as it progresses, and rapid proliferation finally takes place.

The practical deductions to be drawn from such cases are, that we should view all the so-called benign neoplasms, in the light of modern surgical knowledge, as peculiarly liable to undergo not a malignant degeneration but a secondary infection. The soil is prepared for a secondary mixed infection, and we should remove all such growths early and thoroughly, as Doctor Weidner has said.

With further reference to the correlation of germs: We know in what a remarkable degree the tubercle bacillus prepares the soil for pus organism, and we know also in that secondary or mixed infection there are tissue changes due to the tubercle germs, although little impression may be made upon the general system of the patient. The soil is prepared by infection with the tubercle bacillus, and is better suited for the development of other forms of disease. The same thing applies to enchondroma, fibroma, and other so-called innocent neoplasms, and I am led to believe there is a more intimate relationship between the different organisms, of which we have no knowledge at present, than we have hitherto assumed. A study of this correlation would be extremely interesting, and might be a valuable aid in the possible determination of the germ which is probably responsible for the development of sarcoma and other neoplasms.

